# Clinical Value of NGAL, L-FABP and Albuminuria in Predicting GFR Decline in Type 2 Diabetes Mellitus Patients

**DOI:** 10.1371/journal.pone.0054863

**Published:** 2013-01-22

**Authors:** Kuei-Mei Chou, Chin-Chan Lee, Chih-Huang Chen, Chiao-Yin Sun

**Affiliations:** 1 Division of Endocrinology and Metabolism, Department of Internal Medicine, Chang Gung Memorial Hospital, Keelung, Taiwan; 2 Division of Nephrology, Department of Internal Medicine, Chang Gung Memorial Hospital, Keelung, Taiwan; The University of Manchester, United Kingdom

## Abstract

**Objectives:**

Neutrophil gelatinase-associated lipocalin (NGAL) and liver-type fatty acid binding protein (L-FABP) are emerging as excellent biomarkers in the urine and plasma for the early prediction of acute and chronic kidney injury. The aims of this prospective study were to determine the role of albuminuria, and that of serum and urine levels of NGAL and L-FABP as predictors of a decline in the glomerular filtration rate (GFR) in patients with type 2 diabetes.

**Methods:**

A longitudinal cohort study with one hundred forty type 2 diabetic patients was conducted. Serum and urine levels of NGAL and L-FABP, and the urine albumin excretion rate were determined. The correlation between the kidney injury biomarkers and rate of GFR decline was analyzed.

**Results:**

The eGFR of study subjects decreased significantly as the study progressed (86.4±31.1 *vs*. 74.4±27.3 ml/min/1.73 m^2^, *P*<0.001), and the urine albumin excretion rate increased significantly (264.9±1060.3 *vs*. 557.7±2092.5 mg/day, *P* = 0.009). The baseline urine albumin excretion rate and serum L-FABP level were significantly correlated with baseline eGFR (*P*<0.05). The results of regression analysis for the correlations between the rate of eGFR change and the baseline levels of NGAL and L-FABP, and the urine albumin excretion rate showed that only the urine albumin excretion rate was significantly correlated with the rate of eGFR change (standardized coefficients: −0.378; t: −4.298; *P*<0.001).

**Conclusions:**

Tubular markers, such as NGAL and L-FABP, may not be predictive factors associated with GFR decline in type 2 diabetic patients.

## Introduction

Diabetes mellitus is the leading cause of chronic kidney disease (CKD) [1]. The kidney injury is often irreversible when the diabetic nephropathy enters the macroalbuminuria or CKD stages [2]. However, pathologic abnormalities are noted in patients with long-standing diabetes mellitus before the onset of microalbuminuria [3]. Deterioration of renal function can be treated and delayed if renal disease is recognized and treated in a timely manner. Early detection and intervention are critical for treating diabetic nephropathy [4], [5]. Microalbuminuria is an early clinical marker for diabetic nephropathy, which is associated with disease progression to end-stage renal disease and cardiovascular events [6]–[9]. Although albuminuria is widely used and is considered the best clinical marker for renal damage in diabetic patients, several studies have suggested that significant kidney damage can appear before microalbuminuria occurs [10]–[12].

Neutrophil gelatinase-associated lipocalin (NGAL) and liver-type fatty acid binding protein (L-FABP) are emerging as excellent biomarkers in the urine and plasma for the early prediction of acute kidney injury [13], [14]. NGAL is produced by neutrophils, which are markedly induced and released in injured epithelial cells, including kidney tubular cells [15]. L-FABP is expressed in renal proximal tubular cells and is assumed to be shed into urine in response to hypoxia caused by decreased peritubular capillary blood flow [16]. Recent studies have reported that high circulating NGAL levels appear to reflect the chronic inflammatory state of CKD patients [17]. Moreover, subjects with higher baseline NGAL showed a considerably increased risk of worsening renal function [18]. Urinary L-FABP may be a useful clinical biomarker for monitoring chronic glomerular disease, and reflect the clinical prognosis of CKD [16], [19]. However, the clinical application of NGAL and L-FABP in predicting the progression of diabetic nephropathy is still uncertain [20]–[23].

The aims of this prospective study were to determine the role of albuminuria, and serum and urine levels of NGAL and L-FABP as predictors of decline in the glomerular filtration rate (GFR) of patients with type 2 diabetes.

## Methods

### Study Subjects

Prevalent type 2 diabetic patients who attended an outpatient clinic from October 2009 to Novenber 2011 were recruited into the study. The inclusion criterion was adults aged >18 but <80 years. Patients were excluded from the study if they had cardiovascular disease (coronary artery disease, myocardial ischaemia, cerebrovascular disease or peripheral artery disease) in the past 3 months, infections requiring admission in the past 3 months, uncontrolled hypertension, or unwillingness to participate in the study. A total of 140 patients were enrolled into the study and gave their informed written consent. All the study subjects were treated according to the ADA diabetes mellitus treatment guideline [24]. This study adhered to the Declaration of Helsinki and was approved by the Ethics Committee of the Institutional Review Board at Chang Gung Memorial Hospital.

### Measurements

The demographic and clinical laboratory data were collected at baseline and the end of the study. The estimated GFR was calculated using the abbreviated Modification of Diet in Renal Disease equation. The GFR decline rate was calculated with the following equation: (GFR at the study end – baseline GFR)/(baseline GFR x follow-up duration). The urine albumin excretion rate was determined as the albumin amount of a 24-h urine collection. Microalbuminuria was diagnosed based on a 24-hour urine collection (from 30–300 mg/day). A urine albumin level above 300 mg/day was defined as macroalbuminuria. The renal injury markers, NGAL (human NGAL ELISA kit, Abnova Co., CA, US), and L-FABP (human L-FABP ELISA Kit, Hycult Biotech Inc., NL, US), were measured in one venous blood sample and one 24 h urine sample at baseline and at the end of the study. Each NGAL and L-FABP reaction was performed in duplicate, according to product recommendations; the mean value of each reaction was used for further statistical analysis.

### Statistic Analysis

Descriptive statistics are expressed as means ± standard deviation or percentage frequency, as appropriate. Paired *t*-tests were used to compare means of continuous variables. Pearson correlation coefficients were used to test correlations between the GFR and GFR decline rate and other renal injury variables. Multiple regression analysis was used to determine the association between the GFR and GFR decline rate and other renal injury variables. Shapiro-Wilk W test was used to test the distribution of variables. Non-parametric test with Spearman's rank correlation coefficient was used for analyzing the correlation between the renal function and kidney injury variables with non-normal distribution. A *P*-value <0.05 was considered significant (two-tailed). Data were analyzed using the commercially available SPSS 16.0 statistical software program (SPSS, Chicago, IL, US).

## Results

### Baseline Study Characteristics

One hundred forty type 2 diabetic patients were included in this study. The study subjects were followed up for 20.31±2.15 months. The general characteristics and laboratory data of the study subjects are listed in Table 1. Most study subjects had baseline albuminuria less than 300 mg/day and eGFR greater than 60 ml/min/1.73 m^2^ (Table 2 and 3). The eGFR of the study subjects decreased significantly as the study progressed (86.4±31.1 *vs*. 74.4±27.3 ml/min/1.73 m^2^, *P*<0.001); and the urine albumin excretion rate was significantly increased (264.9±1060.3 *vs*. 557.7±2092.5 mg/day, *P* = 0.009). Serum levels of NGAL were significantly increased along the study course (70.0±35.7 vs. 90.6±55.6 ng/ml, *P* = 0.001), but the levels of urine NGAL, and serum and urine L-FABP did not change significantly. Blood pressure, blood sugar and lipid profiles did not change significantly, either (Table 1).

**Table 1:** General characteristics and laboratory data of study subjects.

	Initial (n = 140)	Follow-up (n = 140)	*P*
**Age (year)**	56.6±9.8	58.5±9.8	
**Sex (M/F)**	72/68	72/68	
**DM duration (month)**	86.0±71.2	110.1±71.2	
**SBP (mm Hg)**	136.0±14.3	133.8±16.9	
**DBP (mm Hg)**	75.1±8.9	77.6±9.4	
**BMI (kg/m^2^)**	27.6±4.5	27.7±6.8	
**Fasting sugar (mg/dl)**	150.1±47.9	149.8±45.5	
**HbA1c (%)**	7.8±1.6	7.8±1.4	
**Total cholesterol (mg/dl)**	178.1±38.6	173.0±36.0	
**Triglyceride (mg/dl)**	181.1±134.4	188.8±161.7	
**HDL (mg/dl)**	39.9±11.6	41.7±12.2	
**LDL (mg/dl)**	102.7±39.2	98.0±28.5	
**HS-CRP (mg/dl)**	2.8±6.6	3.1±6.4	
**BUN (mg/dl)**	17.4±8.8	18.5±11.6	
**Creatinine (mg/dl)**	1.2±1.4	1.4±1.3	
**eGFR (ml/min/1.73 m^2^)**	86.4±31.1	74.4±27.3	<0.001
**Urine albumin (mg/day)**	264.9±1060.3	557.7±2092.5	0.009
**Serum NGAL (ng/ml)**	70.0±35.7	90.6±55.6	0.001
**Serum L-FABP (pg/ml)**	6810.3±5450.5	7657.7±4733.2	
**Urine NGAL (ng/ml)**	18.3±58.2	23.3±21.0	
**Urine L-FABP (pg/ml)**	7431.6±13761.8	8445.1±11858.8	
**ACEIs (%)**	21.4	20.7	
**ARBs (%)**	53.6	66.2	0.003
**Statins (%)**	62.3	60.4	
**Eztrol (%)**	9.4	9.4	
**Fibrates (%)**	13.0	11.5	
**Insulin (%)**	18.8	21.8	

Abrreviations:

DM: diabettes mellitus.

SBP: systolic blood pressure.

DBP: diastolic blood pressure.

BMI: body mass index.

HbA1c: glycated hemoglobin.

HDL: high-density lipoprotein.

LDL: low-density lipoprotein.

HS-CRP: highly sensitive C-reactive protein.

eGFR: estimated glomerular filtration rate.

NGAL: neutrophil gelatinase-associated lipocalin.

L-FABP: liver-type fatty acid-binding protein.

ACEI: angiotensin-converting-enzyme inhibitor.

ARB: angiotensin receptor blocker.

**Table 2:** Distribution of albuminuria in study subjects.

Urine albumin	Baseline (n = 140)	End of study (n = 140)	*P*<0.001
**Normal**	52.90%	39.29%	
**Microalbuminuria**	35.00%	37.86%	
**Macroalbuminuria**	12.10%	22.86%	

**Table 3:** Distribution of eGFR in study subjects.

eGFR	Baseline (n = 140)	End of study (n = 140)	*P*<0.001
≧**90**	44.60%	27.34%	
<**90**, ≧**60**	35.25%	46.76%	
<**60**	20.14%	25.90%	

### Association of Baseline GFR with Kidney Injury Variables

**Figure 1:**
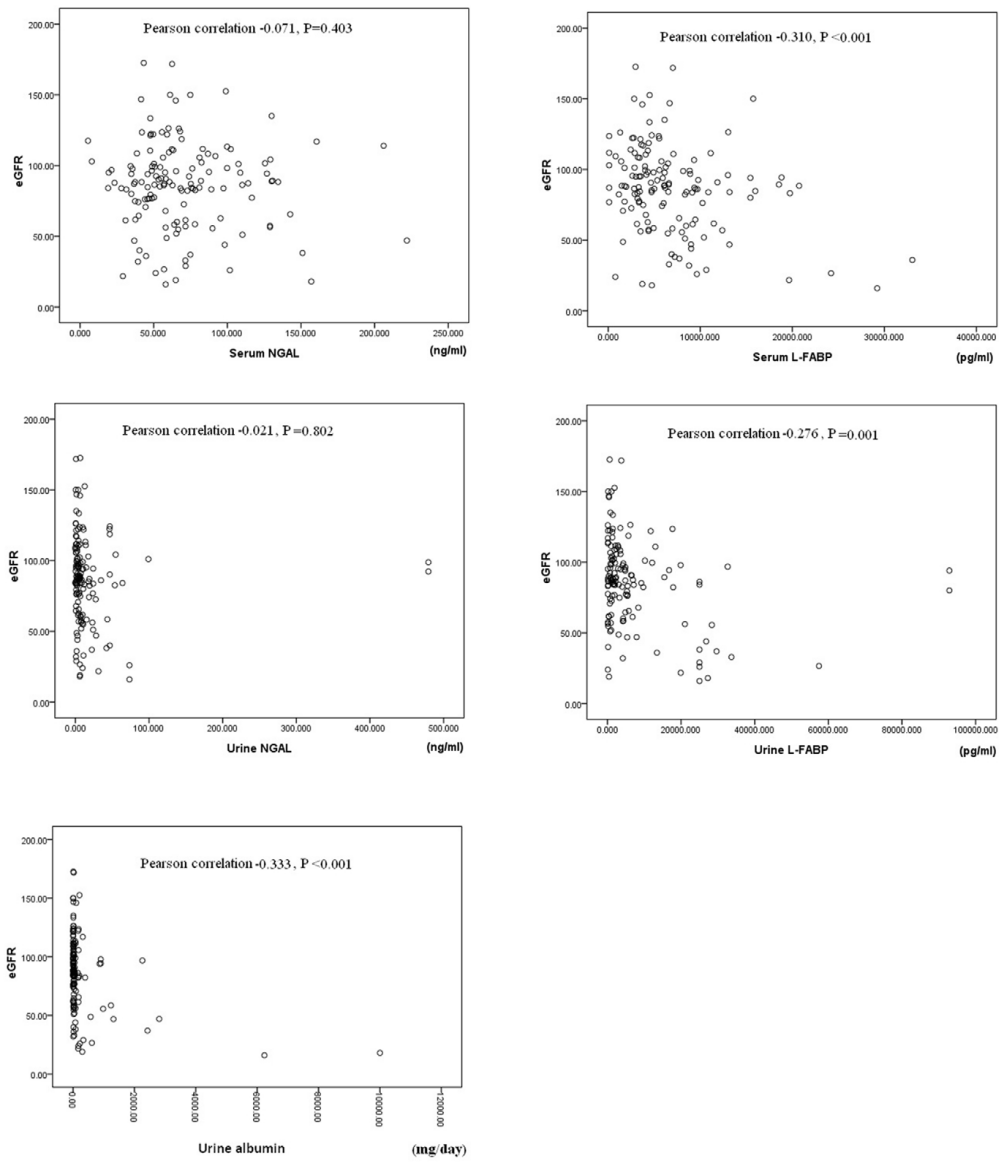
Pearson correlation between baseline eGFR and baseline levels of serum NGAL, serum L-FABP, urine NGAL, and urine L-FABP and the daily urine albumin amount.

The results of Pearson correlation between baseline eGFR and the baseline levels of serum NGAL, serum L-FABP, urine NGAL, and urine L-FABP and the urine albumin excretion rate are plotted in Figure 1. The levels of serum L-FABP (Pearson correlation: −0.310, *P*<.0.001) and urine L-FABP (Pearson correlation: −0.276, *P* = 0.001), and the urine albumin excretion rate (Pearson correlation: −0.333, *P*<0.001), were significantly correlated with the eGFR. The correlations between eGFR and serum/urine L-FABP were not significant.

The results of correlation analysis for the correlations between baseline eGFR and the baseline levels of serum NGAL, serum L-FABP, urine NGAL, and urine L-FABP, and the urine albumin excretion rate are summarized in Table 4 and 5. The baseline urine albumin excretion rate and serum L-FABP level were significantly correlated with baseline eGFR by multiple regression analysis (P<0.05). The correlations between baseline eGFR and serum NGAL, urine NGAL and urine L-FABP levels were not significant (Table 4).

**Table 4:** Correlation between baseline eGFR and the baseline levels of serum NGAL, serum L-FABP, urine NGAL, and urine L-FABP, and the daily urine albumin amount.

Baseline eGFR	Standardized coefficients (beta)	t	*P*
**Urine albumin**	−0.251	−2.985	0.003
**Serum NGAL**	−0.022	−0.278	0.781
**Serum L-FABP**	−0.221	−2.481	0.014
**Urine NGAL**	−0.012	−0.154	0.878
**Urine L-FABP**	−0.126	−1.437	0.153

Multiple regression analysis results. **B:** Spearman's rank correlation coefficient analysis.

**Table 5:** Correlation between baseline eGFR and the baseline levels of serum NGAL, serum L-FABP, urine NGAL, and urine L-FABP, and the daily urine albumin amount.

Baseline eGFR	Spearman's rho	*P*
**Urine albumin**	−0.3416	<0.001
**Serum NGAL**	−0.0152	0.858
**Serum L-FABP**	−0.2814	0.001
**Urine NGAL**	−0.2313	0.006
**Urine L-FABP**	−0.2547	0.002

Spearman's rank correlation coefficient analysis results.

Due to the distribution of baseline levels of serum NGAL, serum L-FABP, urine NGAL, and urine L-FABP and the urine albumin excretion rate were not normal by Shapiro-Wilk W test (P>0.05), Spearman's rank correlation coefficient was calculated. The results showed that baseline urine albumin excretion rate, urine NGAL, and serum/urine L-FABP levels were significantly correlated with baseline eGFR (P<0.05) (Table 5).

### Association of GFR Decline Rate with Baseline Kidney Injury Variables

**Figure 2:**
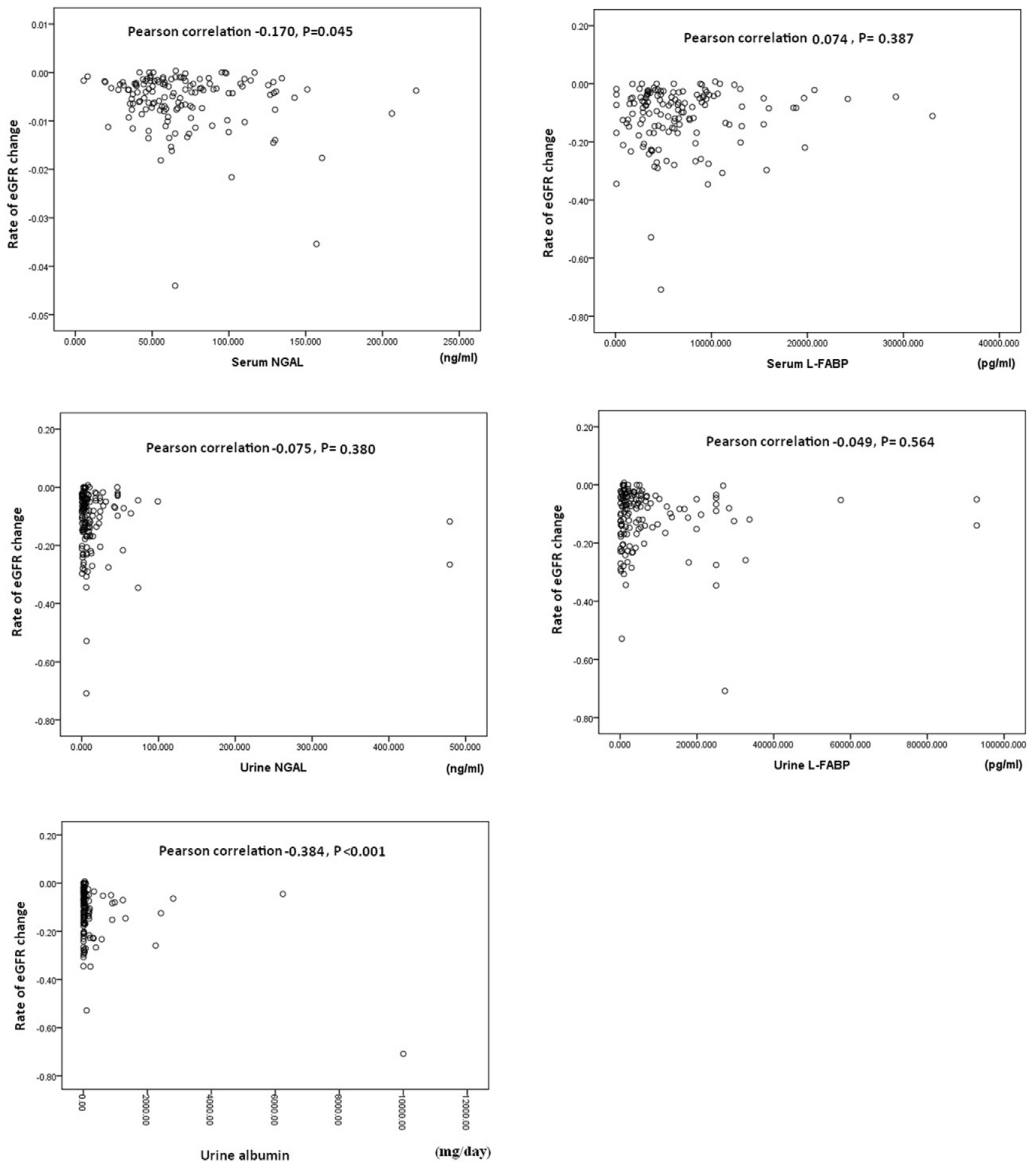
Pearson correlation between the rate of eGFR decline and the baseline levels of serum NGAL, serum L-FABP, urine NGAL, and urine L-FABP, and the urine albumin excretion rate.

Pearson correlations between the rate of eGFR decline and the baseline levels of serum NGAL, serum L-FABP, urine NGAL, and urine L-FABP and the daily urine albumin amount were analyzed (Figure 2). The results showed that the baseline urine albumin excretion rate was significantly correlated with the rate of eGFR decline (Pearson correlation: −0.384, *P*<.0.001). The baseline serum NGAL levels were also correlated with the rate of eGFR decline (Pearson correlation: −0.170, *P* = 0.045). No significant correlation between the rate of eGFR decline and other renal injury markers was noted.

The results of correlations between the rate of eGFR decline and the baseline levels of serum NGAL, serum L-FABP, urine NGAL, and urine L-FABP and the urine albumin excretion rate were summarized in Table 6 and 7. The results of multiple regression analysis showed that only the urine albumin excretion rate was significantly correlated with the eGFR decline rate (standardized coefficients: −0.378; t: −4.298; P<0.001) (Table 6).

**Table 6:** Correlation between the rate of eGFR decline and the baseline levels of serum NGAL, serum L-FABP, urine NGAL, and urine L-FABP, and the urine albumin excretion rate.

Rate of eGFR decline	Standardized coefficients (beta)	t	*P*
**Urine albumin**	−0.378	−4.298	<0.001
**Serum NGAL**	−0.101	−1.238	0.218
**Serum L-FABP**	0.108	1.226	0.222
**Urine NGAL**	−0.061	−0.769	0.443
**Urine L-FABP**	−0.065	−0.726	0.469

Multiple regression analysis results.

**Table 7:** Correlation between the rate of eGFR decline and the baseline levels of serum NGAL, serum L-FABP, urine NGAL, and urine L-FABP, and the urine albumin excretion rate.

Rate of eGFR decline	Spearman's rho	*P*
**Urine albumin**	−0.2732	0.001
**Serum NGAL**	−0.1014	0.233
**Serum L-FABP**	0.0415	0.627
**Urine NGAL**	−0.0367	0.667
**Urine L-FABP**	0.0193	0.821

Spearman's rank correlation coefficient analysis results.

The results of Spearman's rank correlation coefficient analysis for the correlations between the rate of eGFR decline and the baseline levels of kidney injury variables revealed that only the urine albumin excretion rate was significantly correlated with the eGFR decline rate (Spearman's rho: −0.2732; P = 0.001) (Table 7).

The results of subgroup analysis according to the daily urine albumin excretion rate were listed in supplemental tables. In patients with daily urine albumin excretion rate less than 30 mg, all the kidney injury markers were not significantly correlated with the eGFR decline rate (Tables S1 and S2). In patients with daily urine albumin excretion rate greater than 30 mg, urine albumin and serum L-FABP were significantly associated with eGFR decline rate by regression analysis (Table S3). However, only urine albumin was significantly associated with eGFR decline rate by the nonparametric correlation analysis (Table S4).

## Discussion

Diabetic nephropathy is currently the leading cause of CKD. It is also one of the most significant long-term complications in terms of morbidity and mortality for individual patients with diabetes [25]. It is well known that severe tubulointerstitial damage is associated with a faster decline in eGFR in CKD patients [26]. In this study, we used two renal tubular injury biomarkers, NGAL and L-FABP, in addition to albuminuria, to predict the GFR decline rate in type 2 diabetic patients. Our results showed that the serum L-FABP level was significantly associated with eGFR, using regression analysis in the cross-sectional study. However, only the urine albumin excretion rate was significantly associated with eGFR and the eGFR decline rate in type 2 diabetic patients.

Tubulointerstitial and glomerular injuries have important roles in the pathogenesis of diabetic nephropathy [2]. Several recent studies demonstrated that urinary tubular damage markers, such as KIM-1, NGAL and L-FABP, may have the potential to be clinical markers for identifying the development or progression of diabetic nephropathy [27]–[30]. It was also reported that urine NGAL was significantly elevated in type 1 diabetic patients with or without albuminuria, and that urine NGAL increased significantly with increasing albuminuria [27]. However, some studies have shown conflicting results. A study with type 2 diabetic patients showed that the urinary tubular markers, NGAL and L-FABP, were not significantly increased in the normoalbuminuria and microalbuminuria groups, compared to the normal control group [31]. Another study with type 1 diabetic patients revealed that urine NGAL and L-FABP levels were not related to the decline in GFR, after adjustment for known promoters of progression [21]. A matched case-control study for predicting incident CKD stage 3 also showed that adjustment for urinary creatinine and albumin concentration attenuated this association between NGAL and incident CKD stage [32]. Our study included 140 diabetic patients with varying degrees of diabetic nephropathy; however, most of them had mild diabetic nephropathy (albuminuria <300 mg/day; eGFR >60). We observed that albuminuria increased and eGFR decreased in our study subjects as the study progressed. However, except serum NGAL, the urine NGAL and serum/urine L-FABP levels did not change significantly throughout the course of the study. The results of multivariate analysis also showed that NGAL and L-FABP lacked clinical value in predicting the GFR decline rate in type 2 diabetic patients.

Albuminuria is a clinical biomarker for glomerular injury. According to the staging, initial changes in diabetic nephropathy include glomerular hyperfiltration. The phase following hyperfiltration is associated with subtle morphological changes, including thickening of the glomerular basement membrane, glomerular hypertrophy, mesangial expansion, and modest expansion of the tubulointerstitium. In the microalbuminuric phase, significant glomerular injury is often noted. In advanced diabetic nephropathy, nodular glomerulosclerosis is the most prominent pathological presentation [33], [34]. Tubulointerstitial injury in the kidney is considered as a final common pathways to end-stage renal failure [35]. Tubular cell proliferation and tubular hypertrophy are main presentations in the early diabetic nephropathy [36]. Tubulointerstitial fibrosis is the late pathological presentation of chronic kidney disease. It is known that the rate of deterioration of renal function correlates best with the degree of tubulointerstitial fibrosis in diabetic nephropathy. These studies suggest that although in the majority of patients the primary event is a condition manifest by glomerular changes resulting in proteinuria, the long-term outcome is determined by tubulointerstitial fibrosis [37]–[39]. In our study, most study subjects had early-stage diabetic nephropathy in the study beginning. We speculated that relatively less severe tubulointerstitial injury in our patients with early diabetic nephropathy might lead to the failure of the tubular injury markers, NGAL and L-FABP, to early predict the GFR deline in diabetic patients. This might also explain why only albuminuria was significantly correlated with the GFR decline rate in our study.

The results of this study are subject to some limitations. First, the sample number and unhomogenized characteristics of the study subjects might confound the study results. Second, this was a longitudinal observational study. The lack of a control group or effective intervention for comparison might limit the power of the study. Despite these limitations, the results of the cross-sectional analysis with baseline data were compatible with the longitudinal analysis results.

In conclusion, the results of this study suggest that tubular markers, such as NGAL and L-FABP, may not be predictive factors associated with GFR decline in type 2 diabetic patients. In addition, the urine albumin excretion rate was an independent factor associated with GFR decline rate in type 2 diabetic patients.

## Supporting Information

Table S1 **Correlation between the rate of eGFR decline and the baseline levels of serum NGAL, serum L-FABP, urine NGAL, and urine L-FABP, and the urine albumin excretion rate in patients with daily urine albumin excretion rate less than 30 mg.** Multiple regression analysis results.
(DOC)

Table S2 **Correlation between the rate of eGFR decline and the baseline levels of serum NGAL, serum L-FABP, urine NGAL, and urine L-FABP, and the urine albumin excretion rate in patients with daily urine albumin excretion rate less than 30 mg.** Spearman's rank correlation coefficient analysis results.
(DOC)

Table S3 **Correlation between the rate of eGFR decline and the baseline levels of serum NGAL, serum L-FABP, urine NGAL, and urine L-FABP, and the urine albumin excretion rate in patients with daily urine albumin excretion rate greater than 30 mg.** Multiple regression analysis results.
(DOC)

Table S4 **Correlation between the rate of eGFR decline and the baseline levels of serum NGAL, serum L-FABP, urine NGAL, and urine L-FABP, and the urine albumin excretion rate in patients with daily urine albumin excretion rate greater than 30 mg.** Spearman's rank correlation coefficient analysis results.
(DOC)
