# Supplementary material for: Clinical Value of NGAL, L-FABP and Albuminuria in Predicting GFR Decline in Type 2 Diabetes Mellitus Patients
Source: PLoS One. 2013 Jan 22;8(1):e54863. doi: 10.1371/journal.pone.0054863 (PMC3551928; doi:10.1371/journal.pone.0054863)
Supplement: Table S1 — Correlation between the rate of eGFR decline and the baseline levels of serum NGAL, serum L-FABP, urine NGAL, and urine L-FABP, and the urine albumin excretion rate in patients with daily urine albumin excretion rate less than 30 mg. Multiple regression analysis results. (DOC) [file pone.0054863.s001.doc]

**Table S1:**

| **Rate of eGFR decline** | **Standardized coefficients (beta)** | **t** | ***P*** |
| --- | --- | --- | --- |
| **Urine albumin** | 0.114 | 0.757 | 0.452 |
| **Serum NGAL** | -0.206 | -1.64 | 0.106 |
| **Serum L-FABP** | -0.028 | -0.226 | 0.822 |
| **Urine NGAL** | -0.163 | -1.365 | 0.177 |
| **Urine L-FABP** | -0.130 | -0.914 | 0.364 |
