# Supplementary material for: Clinical Value of NGAL, L-FABP and Albuminuria in Predicting GFR Decline in Type 2 Diabetes Mellitus Patients
Source: PLoS One. 2013 Jan 22;8(1):e54863. doi: 10.1371/journal.pone.0054863 (PMC3551928; doi:10.1371/journal.pone.0054863)
Supplement: Table S3 — Correlation between the rate of eGFR decline and the baseline levels of serum NGAL, serum L-FABP, urine NGAL, and urine L-FABP, and the urine albumin excretion rate in patients with daily urine albumin excretion rate greater than 30 mg. Multiple regression analysis results. (DOC) [file pone.0054863.s003.doc]

**Table S3:**

| **Rate of eGFR decline** | **Standardized coefficients (beta)** | **t** | ***P*** |
| --- | --- | --- | --- |
| **Urine albumin** | -0.578 | -4.278 | <0.001 |
| **Serum NGAL** | -0.027 | -0.206 | 0.837 |
| **Serum L-FABP** | 0.353 | 2.228 | 0.031 |
| **Urine NGAL** | 0.120 | 0.994 | 0.350 |
| **Urine L-FABP** | -0.060 | -0.393 | 0.696 |
