# Supplementary material for: Clinical Value of NGAL, L-FABP and Albuminuria in Predicting GFR Decline in Type 2 Diabetes Mellitus Patients
Source: PLoS One. 2013 Jan 22;8(1):e54863. doi: 10.1371/journal.pone.0054863 (PMC3551928; doi:10.1371/journal.pone.0054863)
Supplement: Table S4 — Correlation between the rate of eGFR decline and the baseline levels of serum NGAL, serum L-FABP, urine NGAL, and urine L-FABP, and the urine albumin excretion rate in patients with daily urine albumin excretion rate greater than 30 mg. Spearman's rank correlation coefficient analysis results. (DOC) [file pone.0054863.s004.doc]

**Table S4:**

| **Rate of eGFR decline** | **Spearman's rho** | ***P*** |
| --- | --- | --- |
| **Urine albumin** | -0.463 | 0.001 |
| **Serum NGAL** | -0.148 | 0.299 |
| **Serum L-FABP** | 0.147 | 0.305 |
| **Urine NGAL** | 0.103 | 0.470 |
| **Urine L-FABP** | 0.045 | 0.756 |
